# Recurrence and mortality rate in an Italian multi-center case series of parathyroid atypical adenomas and carcinomas

**DOI:** 10.3389/fendo.2023.1158474

**Published:** 2023-05-08

**Authors:** Marco Barale, Alice Nervo, Andrea Craparo, Alessia Pusterla, Francesca Retta, Federica Maiorino, Elena Castellano, Alessandro Piovesan, Laura Gianotti, Giorgio Borretta, Massimo Procopio, Emanuela Arvat

**Affiliations:** ^1^ Division of Endocrinology, Diabetology and Metabolic Diseases, Department of Medical Sciences, Città Della Salute e Della Scienza Hospital, University of Turin - Cso Dogliotti, Turin, Italy; ^2^ Oncological Endocrinology Unit, Department of Medical Sciences, Città Della Salute e Della Scienza Hospital, University of Turin - Via Genova, Turin, Italy; ^3^ Division of Endocrinology, Diabetology and Metabolic Diseases, Department of Medicine, A. O. Santa Croce e Carle - S.Croce Hospital - Via Coppino, Cuneo, Italy

**Keywords:** parathyroid atypical adenoma, parathyroid carcinoma, recurrence, mortality, ki67, follow up

## Abstract

**Introduction:**

There are few data regarding the clinical outcome of patients with parathyroid carcinoma (PC) and atypical adenoma (AA) after surgery. Aim of our study was to investigate disease recurrence and mortality rate as well as their predictors in a series of patients with PC or AA.

**Methods:**

Clinical and biochemical parameters, histological features, incidence of disease recurrence and mortality rate were retrospectively assessed in 39 patients (51% males, mean age 56.2 ± 17.2 years) diagnosed with PC (n=24) or AA (n=15) and followed up for 6.8 ± 5.0 years after surgery.

**Results:**

No differences in baseline characteristics were registered between the two groups, except for higher KI67 values in PC than AA (6.9 ± 3.9% vs 3.4 ± 2.1%, p<0.01). Eight patients (21%) experienced recurrence after a mean follow-up of 5.1 ± 2.7 years, with higher relapse rate in PC than AA (25% vs 13%), though this difference did not reach statistical significance. Mortality rate was 10% in the whole sample, without significant differences between PC and AA. Relapsing cases had been undergone the most extensive surgery more frequently and they had a higher mortality rate in comparison to non relapsing patients (38% vs 6% and 38% vs 3%, respectively, p<0.03 for both). In comparison to survivors, deceased patients were submitted to the most extensive surgery more frequently (50% vs 9%), they were older (74.8 ± 4.6 vs 53.2 ± 16.3 years), and they had higher KI67 values (11.7 ± 4.9 vs 4.8 ± 2.8, p<0.03 for all comparisons).

**Conclusions:**

During seven-year follow-up after surgery, no significant differences in recurrence and mortality rate were observed between PC and AA patients. Death was associated with disease relapse, older age and higher KI67 values. These findings suggest a similar and careful long-term follow-up in both parathyroid tumors, especially in older patients, and emphasize the need of further studies in large cohorts to throw light on this crucial clinical issue.

## Introduction

Parathyroid carcinoma (PC) and atypical adenoma (AA) are rare causes of primary hyperparathyroidism (PHPT) (1, 2). PC accounts for < 1% of all cases and it is diagnosed in presence of at least one definite criteria of malignancy: angioinvasion, lymphatic invasion, perineural or intraneural invasion, unequivocal invasion into adjacent structures and/or histologically or cytologically confirmed metastases (3). PC is often sporadic but it can occur in the context of familial syndromes, such as hyperparathyroidism-jaw tumor (HPT-JT), multiple endocrine neoplasia type 1 and 2A (MEN 1, MEN2A) and familial isolated hyperparathyroidism (FIHP) (1). Mutations of the tumor suppressor CDC73 gene, encoding a loss-of-function protein termed parafibromin, are responsible for up to 70% of sporadic PC and for HPT-JT-related PC (1). PC is mainly characterized by an indolent course, although recurrences and distant metastases can occur: the former have been observed in up to 50% of patients, while the latter are rarer (1, 4, 5). The term AA is no longer endorsed by the recent 2022 WHO classification of parathyroid tumors, which introduces the category “atypical parathyroid tumor”, meaning a neoplasm with an uncertain potential for malignancy which shows several atypical cytological and architectural features that may also be found in PC, but lacks unequivocal signs of invasion (3). Atypical features include cytologic atypia (atypical mitotic figures), increased mitotic activity (> 5 per 10 mm^2^), Ki67 index > 5%, trabecular growth, fibrosis (including band-like fibrosis), coagulative necrosis, adherence to adjacent structures without invasion and cells extending into but not through the capsule (1, 3). Like PC, also AA may be found in the context of a familial syndrome (2), so that genetic testing, including CDC73 analysis, is recommended in both parathyroid tumors, in order to offer genetic counseling in first-degree relatives. A recent literature revision analyzing 672 patients with AA shows a recurrence rate of 3%, higher in familial than in sporadic cases (40 vs 2%), though significant differences among the included studies have been reported (2). Moreover, discrepant findings about mortality rate in PC and AA have been disclosed (6–10), mostly due to different neck surgery extension.

Although histopathological differences between these two entities are codified, differential diagnosis is still difficult in some cases, also for expert pathologists (2). Moreover, clinical presentation and epidemiological characteristics may overlap, making differential diagnosis even more difficult and leading to speculate that AA could represent an early stage of PC (2). So far, few studies have compared the clinical presentation, the biochemical and histological features as well as the recurrence and mortality rate of PC and AA (11–15). Furthermore, most of these studies did not take into account some biases, as the impact of previous neck surgery extension on disease recurrence.

The aim of our study was thus to investigate the clinical and biochemical features as well as the KI67 values of patients with PC or AA, together with the disease recurrence and the mortality rate, trying to identify some parameters predicting their clinical course after surgery.

## Materials and methods

This multi-center retrospective analysis included data of consecutive adult patients diagnosed with PC or AA and followed at AOU Città della Salute e della Scienza of Turin and AO Santa Croce e Carle of Cuneo (Piedmont, Italy). The study included all patients who underwent parathyroidectomy (PTX), with or without thyroid lobectomy (TL) or total thyroidectomy (TT) and with or without lymph node dissection, from January 2000 until December 2020. Neck surgery extension was based on the detection of moderate-marked hypercalcemia and/or elevated PTH levels and on the aspect of the lesion (such as macroscopic infiltration or adherence to adjacent structures, including controlateral thyroid lobe). None of patients who underwent total thyroidectomy or thyroid lobectomy had a concomitant thyroid disease needing surgical approach per se. Specifically, among patients who underwent total TX, 1 AA and 4 PC had US low-risk micronodular goiter. Histological diagnosis of PC was made in referring Centers by expert pathologists basing on the presence of an invasive growth pattern; conversely, AA cases did not fulfill the criteria of unequivocal malignancy, but exhibited atypical histopathologic features of uncertain malignant potential. Diagnosis were established according to the WHO guidelines in force at the time of patient’s assessment (3).

Data collected at diagnosis were compared between PC and AA patients and included demographic characteristics (gender, age), clinical presentation (evidence of symptomatic renal or bone complications), blood and urine tests (serum calcium, phosphorous, 25-hydroxy vitamin D, creatinine and parathormone (PTH) values, 24-hour urine calcium levels), bone mineral density (T-score at lumbar spine and total hip) and renal ultrasound. Both Centers involved in the study measured PTH values by using a second-generation immunoassay (Diasorin) with the same reference range (i.e. 15-65 pg/ml). Inter- and intra-assay coefficients of variation were 5.5% and below 3%, respectively, while the detection limit of the assay was 3.0 pg/ml. Moreover, regarding the evaluation of bone mineral density, both Centers have an Hologic instrument, with similar technical performance. Diagnosis of chronic kidney failure was based on current guidelines (16) and glomerular filtration rate was calculated according to CKD-EPI formula. Other data including neck surgery extension, post-surgical complications, Ki-67 index, incidence and time of recurrence after the first surgery and mortality rate were recorded. Recurrent disease was defined in case of confirmed detection of elevated serum calcium and PTH levels at least six months after successful parathyroid surgery, with or without radiological evidence of loco-regional disease or distant metastasis. All the variables were compared between patients with or without disease recurrence as well as between deceased and surviving patients.

The study was performed according to the Declaration of Helsinki II and approved by the local Ethics Committees.

Serum total and urinary calcium (mmol/l and mmol/die) and phosphate (mmol/l) were tested using automated methods based on colorimetric and enzymatic assays. Intact PTH (iPTH) samples were tested using a second-generation chemiluminescent immunoassay. Serum 25(OH)vitamin D (nmol/l) was tested by a radioimmunoassay method using an antibody with specificity to 25(OH)vitamin D. Plasma creatinine levels (µmol/l), was measured by enzymatic colorimetric tests. Glomerular filtration rate (GFR) was calculated according to CKD-EPI formula. Lumbar and femoral BMD were assessed by dual energy x-ray absorptiometry (DXA) on a Hologic QDR 4500 instrument (Bedford, MA) and expressed as T-score.

Characteristics of patients were summarized descriptively using mean and standard deviation, or numbers and percentages. Normality of frequency distribution functions was tested by the Shapiro–Wilk W-test. Between-group differences were evaluated by the Chi-square test or Fisher’s exact test for categorical variables and the Mann–Whitney U test for continuous variables. Recurrence-free and overall survival were estimated by Kaplan–Meier method: the observation period started from the surgical treatment until the date of recurrence or death (failures), whichever occurred first, or until the last follow up visit (censoring). Differences between PC and AA groups were evaluated by the log-rank test. Calculations were performed using SPSS Windows release 24.0. A p-value < 0.05 was considered statistically significant.

## Results

Thirty-nine patients with parathyroid neoplasia were identified, including 24 patients with PC and 15 patients with AA. Baseline clinical, biochemical and histological features of the studied patients are shown in **Table 1**, stratified as follows: PC and AA; relapsing (R+) and non-relapsing (R-); deceased (D) and surviving (S). No significant differences in age, sex, bone metabolism parameters as well as prevalence of renal and skeletal complications were found between PC and AA. Only Ki67 tested on histological samples showed significant higher values in the former than in the latter group (6.9 ± 3.9 and 3.4 ± 2.1% respectively, p<0.01). One patient with PC showed distant bone metastasis at the time of diagnosis.

**Table 1:** Baseline characteristics and incidence of post-surgery hypocalcemia, chronic hypoparathyroidism, disease relapse and death of the studied patients stratified as follows: parathyroid carcinomas (PC) and atypical adenomas (AA); relapsing (R+) and non-relapsing (R-); deceased (D) and surviving (S).

	PC n= 24	AA n = 15	R+ n = 8	R- n = 31	D n = 4	S n =35	Normal Range
Age (years)	57.6 ± 16.2	54.0 ± 19.2	55.5 ± 19.3	55.5 ± 16.5	74.8 ± 4.6 ^b^	53.2 ± 16.3	—
Sex (M)	12 (50)	8 (53)	5 (63)	15 (48)	2 (50)	18 (51)	—
Calcium (mmol/L)	3.49 ± 0.52	3.39 ± 0.77	3.37 ± 0.75	3.47 ± 0.62	3.07 ± 0.32	3.52 ± 0.65	2.2 – 2.6
PTH (pg/mL)	889 ± 653	932 ± 731	853 ± 440	916 ± 714	942 ± 288	902 ± 714	15 – 65
Phosphorous (mmol/L)	0.65 ± 0.23	0.81 ± 0.29	0.84 ± 0.13	0.71 ± 0.29	0.67 ± 0.23	1.10 ± 0.23	0.81 – 1.45
25OHVitD (nmol/L)	38.7 ± 24.2	31.7 ± 15.0	21.0 ± 6.5	39.7 ± 21.0	38.2 ± 20.7	22.0 ± 9.5	75 – 125
24hCalciuria (mmol/die)	9.5 ± 2.0	10.8 ± 2.2	9.8 ± 1.6	10.1 ± 2.1	9.9 ± 1.9	10.1 ± 2.1	2.5 – 7.5
Creatinine (µmol/L)	97 ± 35	132 ± 79	141 ± 0	97 ± 53	141 ± 0	106 ± 53	44 – 106
CKD stage III or higher	11 (46)	9 (60)	5 (63)	15 (48)	2 (50)	18 (51)	—
Nephrolithiasis/calcinosis	10 (42)	4 (27)	3 (38)	11 (35)	1 (25)	13 (37)	—
Lumbar T-score	-3.1 ± 1.4	-2.7 ± 1.3	-2.8 ± 1.4	-2.9 ± 1.3	-2.8 ± 1.2	-2.9 ± 1.4	-1 – +2
Total Femoral T-score	-2.4 ± 0.5	-2.8 ± 2.3	-2.6 ± 0.5	-2.5 ± 0.9	-2.6 ± 0.7	-2.5 ± 1.1	-1 – +2
Fragility fractures	7 (29)	5 (33)	3 (38)	9 (29)	2 (50)	10 (29)	—
Ki67 (%)	6.9 ± 3.9 ^a^	3.4 ± 2.1	7.0 ± 6.1	5.0 ± 2.9	11.7 ± 4.9 ^b^	4.8 ± 2.8	—
Post-surgery hypoCa	8 (33)	10 (67)	4 (50)	14 (45)	3 (75)	15 (43)	
Chronic hypoPTH	2 (8)	3 (20)	1 (13)	4 (13)	0	5 (14)	
Disease relapse	6 (25)	2 (13)	—	—	3 (75)	5 (14)	
Death	3 (13)	1 (7)	3 (38)	1 (3)	—	—	

Data are presented as mean ± standard deviation or n (percentage).

Statistical analysis is performed by Pearson’s X^2^ and Mann–Whitney test.

^a^ p < 0.01 vs AA; ^b^ p < 0.03 vs S.

PTH, parathormone; CKD, chronic kidney disease; hypoCa, hypocalcemia; hypoPTH, hypoparathyroidism. "-" means "not applicable".

**Figure 1:**
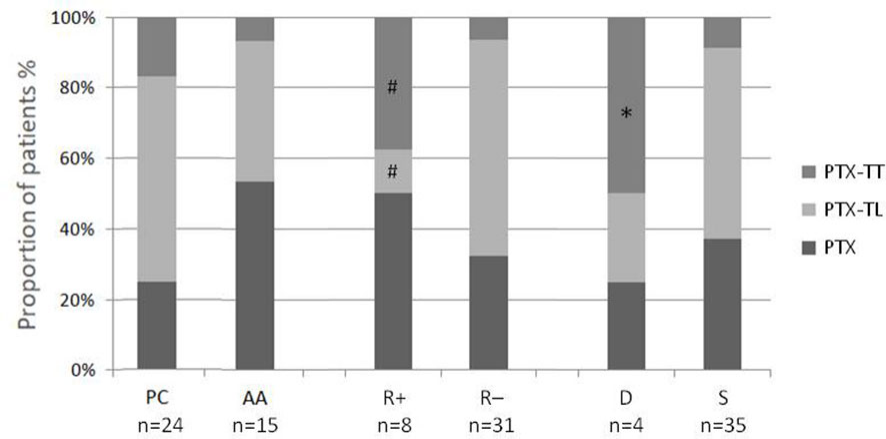
Proportion of patients with various neck surgery extension stratified as parathyroid carcinomas (PC) and atypical adenomas (AA), relapsing (R+) and non-relapsing patients (R-), deceased (D) and surviving patients (S). PTX, simple parathyroidectomy; TL, thyroid lobectomy; TT, total thyroidectomy. ^#^p< 0.03 vs R-; *p< 0.03 vs S.

As depicted in **Figure 1**, neck surgery extension was not significantly different between PC and AA patients, although a trend toward a more extensive surgery was observed for neoplasms come out as PC. Specifically, 13 patients with PC and 5 with AA showing marked hypercalcemia and/or considerably elevated PTH levels, as well as 5 patients with PC and 2 with AA displaying intra-operative signs of macroscopic infiltration or adherence to adjacent structures, underwent a more extensive neck surgery. Among them, 5 PC and 2 AA underwent lymph node dissection of central compartment, without detection of metastatic localization (data not shown).

Eighteen patients (46%) developed post-surgical transient hypocalcaemia while chronic hypoparathyroidism occurred in five patients (13%, specifically 2 PC and 3 AA), requiring treatment with calcitriol and calcium supplements at a mean starting dosage of 0.92 ± 0.63 µg/die and 1510 ± 1446 mg/die, respectively. Specifically, hypoparathyroidism occurred in 2 PC and 1 AA who underwent PTX + TT, and in 2 AA who underwent PTX + TL and concomitant lymph node dissection of central compartment. Moreover, a hungry bone syndrome was reported in 4 patients (10%).

Mean follow up after surgery was 6.8 ± 5.0 years in the whole group, without significant differences between patients with PC (7.4 ± 5.9 years) and AA (6.0 ± 3.4 years, p=0.66).

**Figure 2:**
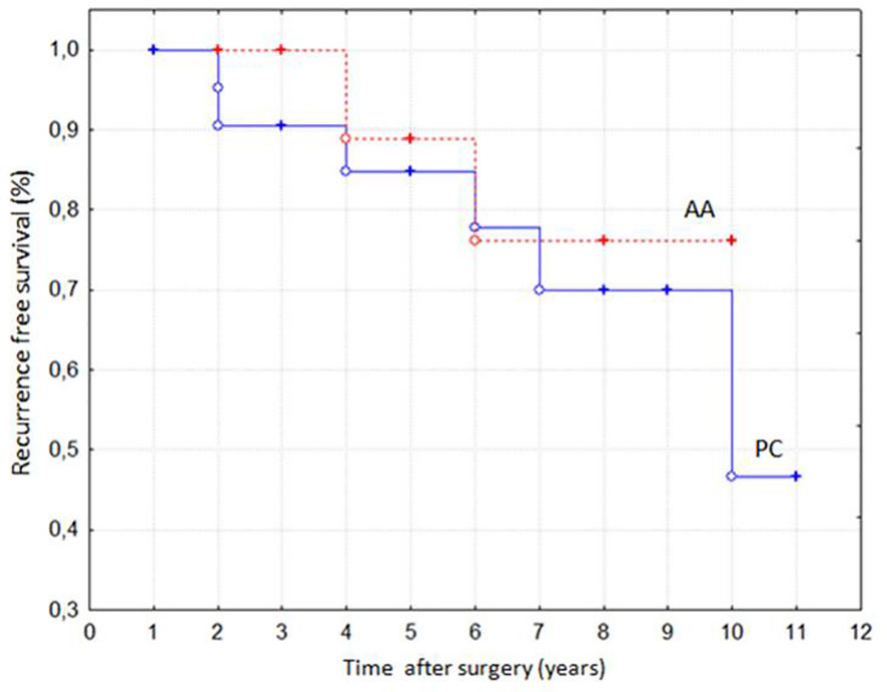
Recurrence-free survival in patients with parathyroid carcinomas (PC) and atypical adenomas (AA) according to Kaplan-Meier analysis (p = NS).

Eight patients (21%) developed disease relapse at the same location of previous surgery, with a higher, although not significant, recurrence rate in PC (n=6, 25%) compared to AA (n=2, 13%). The disease relapse occurred at a similar mean time in PC and AA (5.0 ± 1.4 years vs 5.2 ± 3.1 years). Accordingly, the Kaplan-Meier analysis displayed a similar recurrence-free survival in PC and AA (**Figure 2**). As reported in **Table 1**, no differences were found in baseline clinical and biochemical features as well as in KI67 values between disease-free (R-) and relapsing patients (R+). Moreover, prevalence of both post-surgical transient hypocalcaemia and chronic hypoparathyroidism did not differ between the two groups (R+ and R-, data not shown). However, as depicted in **Figure 1**, the R+ group included a higher proportion of patients who had been undergone PTX + TT (38%) and a lower proportion of patients who had been undergone PTX + TL (13%) in comparison to the R- group (6% and 61%, respectively, p< 0.03 for both comparisons). In addition, none of the patients undergoing lymph node dissection experienced disease relapse (data not shown).

Five relapsing patients with PC required treatment with calcium-mimetic cinacalcet (at mean dosage of 174 ± 58 mg/die at the last visit of follow up), while one of them experiencing refractory hypercalcemia needed therapy with denosumab and temozolomide (at dosage of 200 mg/die for five days a week). Treatment of one patient with temozolomide followed the detection of neuroendocrine features at histopathological analysis of the parathyroid lesion. Moreover, five relapsing patients with PC underwent repeated surgery, achieving disease control, but three of them further relapsed.

During the follow up, four patients (three PC and one AA) died (mortality rate: 10%), without significant differences in overall survival between the two groups (median time: 6.0 and 3.5 years, respectively). All the deaths were related to the parathyroid disease and specifically to the systemic complications of severe hypercalcemia. As shown in **Table 1** and **Figure 1**, comparing deceased and surviving patients, we observed significant differences in terms of age (74.8 ± 4.6 and 53.2 ± 16.3 years, respectively, p<0.01), Ki67 levels (11.7 ± 4.9 and 4.8 ± 2.8%, respectively, p<0.03) and neck surgery extension (frequency of PTX + TT: 50% vs 9%, p<0.03). The mortality rate was higher in relapsing than in disease-free patients (38% vs 3%, p<0.01).

## Discussion

In this multi-center retrospective case series of parathyroid tumors, including PC and AA, the recurrence and the mortality rate are 21 and 10%, respectively, with mortality being associated with disease relapse, older age and higher KI67 values. Despite the histological distinction, the clinical and biochemical presentation as well as the incidence of disease relapse and death during seven-year follow-up after surgery were not significantly different between PC and AA.

Inconclusive data about clinical and biochemical differences between PC and AA have been reported so far (1, 2, 11, 13, 17, 18). In our series, we found a similar clinical presentation in PC and AA, with prevalence of bone and renal involvement being comparable between the two groups, but significantly higher than that reported in patients with primary hyperparathyroidism due to parathyroid adenoma (17, 18). In line with our data and other previous studies (1, 2, 17), Quinn et al. (11) reported no clinical differences at diagnosis between 18 PC and 34 AA from a high-volume New England referral center. Similarly, Fernandez-Ranvier et al. (13) did not report any significant difference in the clinical manifestations in patients with PC and AA at the time of initial diagnosis, except from palpable neck mass more often described in PC. Conversely, in a recent paper, Chen et al. (19), reported that patients with AA presented clinical characteristics much less severe than PC. However, the same Authors showed that prevalence of nephrolithiasis/calcinosis and fragility fractures, as well as BMD values at lumbar and femoral sites were not significantly different between the two groups.

Concerning the biochemical presentation, our patients showed marked hypercalcemia associated with PTH levels higher than 10 times the upper normal range. Only few studies on small series compared biochemical features of PC and AA (11–14, 19), reporting in the majority, though not all of them (11, 19), similar PTH and calcium levels. Accordingly, we found no differences in the biochemical profile of the studied groups, indicating that PTH and calcium levels are not able to discriminate between PC and AA.

In order to improve the diagnostic accuracy, the expression of KI67, a well-known proliferative index used in several human cancers, could be evaluated, though its clinical usefulness has to be proved in this specific setting. In fact, previous studies (11, 12) reported no differences in the KI67 values between PC and AA, thus questioning its value in this setting, even if a trend toward higher levels in PC was reported. As found by other Authors (14, 15, 20), in our series KI67 values resulted significantly higher in PC than AA.

Recurrences are common in PC, occurring in more than a half of patients after a follow-up period of few years, although longer disease-free interval has been reported (1). Conversely, AA showed a relapse rate ranging from 0 to 10%, as reported in already published case series, with some differences between sporadic and familial AA (17, 19, 21). In our series, the incidence of disease relapse in PC was largely lower than those previously reported in other studies (11–15), probably due to an extensive neck surgery rate. In fact, recent guidelines suggested an en-bloc resection by an experienced surgeon in all patients with suspicious of malignancy (22), as it plays a preventive role in recurrent disease. Also a recent paper reported such a surgical approach to be followed by a 13 times lower risk for relapse compared with all the other surgical techniques (23). Conversely, in our study, patients who had been operated on PTX + TT had the highest incidence of relapse. This apparently paradoxical finding could be due to a higher suspicious of malignancy in these patients, on the basis of intra-operative clues and/or the detection of moderate-marked hypercalcemia and/or elevated PTH levels, leading to a more radical intervention, confirmed by the diagnosis of 4 PC among 5 patients who underwent PTX + TT. In our study, among patients with and without relapse, no other differences in clinical, biochemical and histological features were found. Accordingly, previous studies (11–13, 17) failed to identify potential predictive factors of disease recurrence in PC and AA.

Comparing recurrence rate between PC and AA, some previous studies (11, 13, 19) reported higher incidence in the former than in the latter, while other Authors (12) showed no differences in loco-regional relapse rate between patients with PC and AA in eight-year follow up. In our series, we found a higher recurrence rate in PC than in AA, though this difference did not reach a statistical significance. Probably, the small sample sizes together with different duration of follow up could explain these discrepant results. Nevertheless, local recurrence is not negligible in AA, suggesting a similar and careful long-term follow-up in both parathyroid tumors.

A five and ten year survival rates between 77–100% and 49–91%, respectively, have been reported in PC (6–8), whereas data derived from longitudinal retrospective studies in AA indicated an overall survival up to 93% after a follow up of five and ten years (9, 10). Similarly, the mortality rate of our case series was 10%, without significant differences between PC and AA, at seven year follow-up after surgery. Notably, patients who died showed higher recurrence rate, were older and had significantly higher KI67 values than the survived ones. Accordingly, previous studies reported young age as associated with improved survival (24, 25) and KI67 as a valuable negative prognostic factor in patients with PC (26). Finally, in line with our findings, Stojadinovic et al. (14) reported disease recurrence to be associated with a significant decrease in disease specific survival.

Our study has some limitations including: 1) its retrospective design; 2) the small sample size; 3) the lack of some anatomical pathology findings including gland size, trabecular growth, fibrosis, necrosis, loss of expression of parafibromin; 4) the inability to distinguish between familial and sporadic diseases 5) the lack of data about somatic loss-of-function mutations of the CDC73 tumor suppressor gene, encoding parafibromin, that may be associated with some clinical outcomes, including disease recurrence.

Nevertheless, this multi-center seven-year follow up study of PC and AA was able to show a low recurrence and mortality rate in both tumors after surgery, with death associated with disease relapse, old age and high KI67 values.

Further prospective studies with a careful and long-term follow up in both parathyroid tumors are needed.

## Data availability statement

The raw data supporting the conclusions of this article will be made available by the authors, without undue reservation.

## Ethics statement

The studies involving human participants were reviewed and approved by Comitato Etico Interaziendale A.O.U. Città della Salute e della Scienza di Torino - A.O. Ordine Mauriziano - A.S.L. Città di Torino. The patients/participants provided their written informed consent to participate in this study.

## Author contributions

Conceptualization: EA, LG, MP, MB; Methodology: EA, LG, MP, MB, AN; Software: MB; Validation: EA, LG, MP, GB, APi; Formal Analysis: MB, MP, AN, FR, APu, FM, AC; Investigation: MB, AN, FR, APu, FM, AC; Resources: MB, AN, FR, APu, FM, AC; Data Curation: MB, AN, FR, APu, FM, AC; Writing: MB, AN, FR, APu, FM, MP, LG, EC; Writing – Review & Editing: MB, AN, FR, APu, FM, MP, LG, EC; Supervision: EA, LG, MP, APi, GB. Each Author has approved the submitted version and agrees to be personally accountable for the author’s own contributions and for ensuring that questions related to the accuracy or integrity of any part of the work, even ones in which the author was not personally involved, are appropriately investigated, resolved, and documented in the literature. All authors contributed to the article and approved the submitted version.
